# Role of rapid urease test and histopathology in the diagnosis of Helicobacter pylori infection in a developing country

**DOI:** 10.1186/1471-230X-5-38

**Published:** 2005-11-25

**Authors:** Javed Yakoob, Wasim Jafri, Shahab Abid, Nadim Jafri, Zaigham Abbas, Saeed Hamid, Muhammad Islam, Kashif Anis, Hasnain Ali Shah, Hizbullah Shaikh

**Affiliations:** 1 Department of Gastroenterology and Pathology, Aga Khan University Hospital, Karachi, Pakistan

## Abstract

**Background:**

The aim of this study was to determine the effect of commonly self-prescribed proton pump inhibitors (PPI) on the results of rapid urease test and histology for the diagnosis of *H. pylori *infection.

**Methods:**

One hundred-nine consecutive patients with dyspeptic symptoms attending the endoscopy suite were enrolled in this study. Antrum biopsy specimens were collected at endoscopy for the rapid urease test (Pronto Dry, Medical Instrument Corp, France) and histopathology. Sensitivity, specificity, positive predictive value (PPV), negative predictive value (NPV) and like-hood ratio of a positive and negative of Pronto Dry test were compared against histology. The gold standard test for the diagnosis of *H. pylori *infection was histopathology.

**Results:**

Sixty-one percent (66/109) patients were males with mean age of 43 ± 14.1 years and age range 17–80 years. Fifty-two percent (57/109) were not on any medications while 48% (52/109) used PPI before presentation to the outpatients. Pronto Dry was positive in 40% (44/109) and negative in 60% (65/109). Histopathology was positive for *H. pylori *in 57% (62/109) and negative in 43% (47/109). The sensitivity, specificity, PPV, NPV and like-hood ratio of a positive and negative Pronto Dry test with and without PPI were 43.3%, 86.4%, 81.3%, 3.18, 0.656 and 52.8% vs 71.9%, 80%, 82.1%, 69%, 3.59 and 0.35.

**Conclusion:**

This study shows that the sensitivity, specificity, NPV and PPV of rapid urease test was reduced in patients who are on PPI. The exclusive use of the rapid urease test for the diagnosis of *Helicobacter pylori *cannot be recommended in patients with prior PPI use.

## Background

*Helicobacter pylori *(*H. pylori*) infection occurs worldwide. It results in chronic gastritis, ulcer, mucosal associated lymphomas and gastric carcinomas [1,2]. The diagnostic methods available for detecting *H. pylori *infection include serology (IgG ELISA), rapid urease test, histopathology, 13 C-urea breath test (UBT) and polymerase chain reaction (PCR) [3-5]. Rapid urease test is highly specific for *H. pylori *infection and is commonly used for the detection of *H. pylori *infection at endoscopy. It requires a high density of bacteria, and anything that reduces the bacterial load may produce false-negative tests. The diagnostic yield of rapid urease test is enhanced by increasing the number of biopsies taken and the number of sites in the stomach that are biopsied [6]. The sensitivity of urease test is reduced in patients who are taking proton pump inhibitors (PPI), antibiotics or bismuth compounds [7,8]. Any antibiotic active against *H. pylori *will cause a reduction in the numbers of bacteria in the stomach [9]. Also, if the patient has received a drug that reduces the acid in the stomach and raises the pH, this will affect the area of the stomach to be biopsied [10]. H_2_-receptor antagonists (ranitidine and cimetidine) raise the gastric pH, but PPI such as omeprazole and lansoprazole, raise the gastric pH to a higher level. Proton pump inhibitors are known to decrease the activity of *H. pylori *within the stomach and to shift their distribution proximally [8]. H_2_-receptor antagonists differ from proton pump inhibitors as high intragastric pH may cause a reduction in urease activity, unrelated to a reduced bacterial load [11]. This effect may reduce the sensitivity of histological examination and rapid urease testing for *H. pylori *on biopsies taken from recommended sites [8]. In Pakistan a third world country self-prescription is common and medications are available on counter of pharmacies for sale without prescriptions [12,13]. Data from 66 pharmacies evaluated 1231 over-the-counter (OTC) encounters, of which 43% were instances of self-medication, while the rest were given on the advice of pharmacy staff [12]. Self-medication increased with the level of socioeconomic status [13]. Proton pump inhibitors are much cheaper than anti-H_2 _receptor blockers (H_2_-RB) costing as much as 10 cents/pill. In a local tertiary care hospital the prescriptions for PPI in 2003 alone numbered 31086 and 399189 tablets/injections were dispensed on prescriptions. The aim of this study was to determine the effect of commonly self-prescribed proton pump inhibitors (PPI) on the results of the rapid urease test (Pronto Dry) and histology.

## Methods

One hundred-nine consecutive patients with dyspeptic symptoms attending the endoscopy suite of gastroenterology section of Aga Khan University Hospital, Karachi, Pakistan from April 2004–January 2005 were enrolled. There were sixty-six males and forty-three females (age range 17–80 years, mean age 40.89 ± 12 years; Table 1). Clinical symptoms at the time of presentation, diagnosis, drug treatment dosage and duration were noted with endoscopic findings. An informed consent was taken from all patients and study was approved by the ethics review committee. Four antral biopsy specimens were collected at endoscopy from each patient two each for the Pronto Dry (a commercially manufactured rapid urease test by Medical Instrument Corp, France) and histopathology. Pronto Dry consists of a dry filter paper containing urea, phenol red (a pH indicator), buffers and a bacteriostatic agent in a sealed plastic slide. If the urease enzyme of *H. pylori *is present in an inserted tissue sample, the resulting decomposition of urea causes the pH to rise and the color of the dot turns from yellow to a bright magenta. Pronto Dry results were read in 30 minutes and one hour after sampling as directed by the manufacturer. The color change from yellow to pink was considered positive result and no color change as negative for Pronto Dry. In this study sensitivity, specificity, positive predictive value, negative predictive value and accuracy of Pronto Dry were compared against histology in the presence and absence of PPI. Histopathologist was kept blinded about the results of Pronto Dry. Two gastric antral biopsy specimens for histopathology were stained with Hematoxylin and eosin stain for the detection of *H. pylori *and degree of gastritis. In doubtful cases Giemsa staining was carried out, to ascertain presence of *H. pylori*. The degree of gastritis as determined on Hematoxylin and eosin (H & E) stain was scored in accordance with the Sydney system, representing absence of gastritis and minimal, mild, moderate chronic active and severe chronic active gastritis, respectively [14].

### Statistical Analysis

Data was entered and analyzed in Statistical Package for Social Sciences (SPSS) ver13.0. Results are presented as mean ± standard deviation for quantitative variables and number (percentages) for qualitative variables. Sensitivity, specificity, positive and negative predictive values of urease test was calculated by two by two standard method. Likelihood ratio of a positive test was equal to sensitivity/(1-specificity) and of a negative test was (1 - sensitivity)/specificity.

## Results

There were 57% (66/109) males and 43% (43/109) females with age range 17–80 years and mean age of 43 ± 14.1 years (Table 1). 52% (57/109) were not on any medications while 48% (52/109) used PPI before presentation to the outpatients.

### Clinical Features

Abdominal pain was present in 75% (82/109), dyspepsia 8% (9/109), vomiting 6% (7/109), heartburn in 6% (7/109), and weakness 5% (4/109) (Table 1). The main endoscopic findings were gastritis 65% (71/109), gastroesophageal reflux disease 16% (17/109) and duodenitis in 10% (11/109) (Table 1).

### Urease test

Pronto Dry was positive in 40% (44/109) and negative in 60% (65/109) (Table 1). The sensitivity, specificity, NPV and PPV of Pronto Dry with and without PPI was 43.3%, 86.4%, 81.3% and 52.8% vs. 71.9%, 80%, 82.1% and 69% (Table 2).

### Histopathology

Histopathology was positive for *H. pylori *in 57% (62/109) and negative in 43% (47/109) (Table 1).

### Comparison of Urease test and Histopathology

The sensitivity, specificity, positive predictive value (PPV) and negative predictive value (NPV) of urease test was reduced on PPI (Table 2). The likelihood of a positive urease test with and without PPI was 3.18 and 3.59 and negative urease test with and without PPI was 0.65 and 0.35.

## Discussion

The rapid urease test is the most frequently used test for the diagnosis of *H. pylori *infection in routine gastrointestinal endoscopy practice. It is extremely valuable because it gives a positive result for *H. pylori *infection before the patient leaves the endoscopic suite. Histological diagnosis of *H. pylori *infection is usually reserved for patients with a negative biopsy urease test or when histology was required for another reason such as exclusion of malignancy. In an earlier study rapid urease test (Pronto Dry) had the sensitivity, specificity, positive predictive value, negative predictive value and diagnostic accuracy were 98%, 100%, 100%, 98% and 99%, respectively [15]. The sensitivity and specificity of Pronto Dry against culture were 98% and 97% [16].

In this study treatment with a PPI before endoscopy reduced the sensitivity of urease test from antral biopsies for *H. pylori *detection. Ideally PPI should be discontinued before the endoscopy [8]. However, in our practice patients quite frequently self medicate. Even referrals from primary care service cannot be discontinuing PPIs for an adequate period before endoscopy. In patients on PPI the biopsy specimen may contain low bacterial density of viable cells giving a negative urease test. This also leads to lack of *H. pylori *identification on histology. Of the various tests that are available for *H. pylori *detection, histological examination of gastric biopsy is considered the most accurate method of diagnosis [6]. In a previous study even histological examination sensitivity, specificity, positive predictive value, negative predictive value and diagnostic accuracy were demonstrated to be reduced on acid reducing drugs [8]. It was demonstrated that after 4 weeks of omeprazole treatment, the histological density of *H. pylori *in the antrum and corpus was reduced, while that in the fundus was increased [17]. The migration of *H. pylori *from the antrum to the fundus was also associated with a corresponding decrease in the activity of antral gastritis and matched by a progressive fall in the excretion of ^13^C urea breath test [17]. In an animal model of *H. pylori *infection antrum-body transitional zone was identified as a sanctuary site in treatment failure [18]. If more than one gastric biopsy tissue is used to inoculate the rapid urease test a positive test might appear thus improving the test sensitivity without compromising its specificity. The diagnostic yield is said to be increased by over 5% by taking more than a single biopsy [6]. However, this also prolongs the endoscopy time. Our study showed the likelihood of a negative urease test with and without PPI was 0.65 and 0.35, respectively. Hence, in patients on PPI additional biopsies should be taken from the body of the stomach beside the antrum for the detection of *H. pylori*. This will be consistent with previous studies which recommend obtaining biopsies both from antrum and body of the stomach in patients on PPIs for the diagnosis of *H. pylori *infection [6,8]. This is also important in view of the high prevalence of *H. pylori *in the region. As rapid urease test can miss a low-level infection with *H. pylori*, a negative test should not be the sole criterion for either absence or cure of *H. pylori *infection. A negative diagnosis on PPI might be backed up with a serological test which should not be affected by PPI. Also, in view of the small sample size of our study, the result needs to be confirmed in a larger population of patients. In conclusion it is of particular relevance to know if a PPI has been used before the patient undergoes diagnostic endoscopy. If PPI can not be discontinued for an adequate period before the endoscopy multiple biopsies should be taken from both the antrum and the body of the stomach for *H. pylori *detection.

## Competing interests

The author(s) declare that they have no competing interests.

## Authors' contributions

JY conceived, designed and coordinated the study, WJ, SA, NJ, ZA, SH, HAS, KA, HS participated in its design and coordination MI participated in the design of the study and performed the statistical analysis. All authors read and approved the final manuscript.

## Pre-publication history

The pre-publication history for this paper can be accessed here:



## Figures and Tables

**Table 1:** Clinical, endoscopic and histopathology findings of the patients (n = 109)

**Factors**	**With PPI n (%)**	**Without PPI n (%)**
**Gender**		
Male	35 (53)	31 (47)
Female	17 (40)	26 (60)
		
**Age (in years) mean ± SD**	42 ± 12.2	44 ± 15.6
		
**Clinical feature**		
Abdominal pain	44 (54)	38 (46)
Heart burn	4 (57)	3 (43)
Vomiting	2 (29)	5 (71)
Dyspepsia	1 (11)	8 (89)
Weakness	1 (25)	3 (75)
		
**Endoscopic findings**		
Gastritis	36(51)	35(49)
Gastritis and GERD	2(100)	-
GERD	6(35)	11(65)
Gastric Ulcer	-	3(100)
Duodenitis	7(64)	4(36)
Duodenal Ulcer	1(20)	4(80)
		
**Urease Test**		
Positive	16 (36)	28 (64)
Negative	36 (55)	29 (45)
		
**Histopathology**		
Positive	30 (48)	32 (52)
Negative	22 (47)	25 (53)

Results are presented as mean ± standard deviation and number (percentages).

SD = Standard Deviation

**Table 2:** The comparison of Urease test and histopathology with the presence and absence of PPI.

**Test**	**Sensitivity (95% C.I.)**	**Specificity (95% C.I.)**	**PPV (95% C.I.)**	**NPV (95% C.I.)**
Urease Test with PPI	43.3% (30.9–50.6%)	86.4% (69.5–96.2%)	81.3% (58–94.8%)	52.8% (42.4–58.3%)
Urease Test without PPI	71.9% (59.2–80.7%)	80% (63.7–91.3%)	82.1% (67.6–92.3%)	69% (55–78.7%)

*95% confidence interval (CI) are given in brackets

## References

[B1] Blaser MJ (1997). Ecology of *Helicobacter pylori *in the human stomach. J Clin Invest.

[B2] Blaser MJ, Perez-Perez GI, Kleanthous H, Cover TL, Peek RM, Chyou PH, Stemmermann GN, Nomura A (1995). Infection with *Helicobacter pylori *strains possessing cagA is associated with an increased risk of developing adenocarcinoma of the stomach. Cancer Res.

[B3] Goossens H, Glupczynski Y, Burette A, Van den Borre C, Butzler JP (1992). Evaluation of commercially available second generation immunoglobulin G enzyme immunoassay for detection of *Helicobacter pylori *infection. J Clin Microbiol.

[B4] Klein PD, Malaty HM, Martin RF, Graham KS, Genta RM, Graham DY (1996). Noninvasive detection of Helicobacter infection in clinical practice: The 13C urea breath test. Am J Gastroenterol.

[B5] Brooks HJ, Ahmed D, McConnell MA, Barbezat GO (2004). Diagnosis of *Helicobacter pylori *infection by polymerase chain reaction: is it worth it?. Diagn Microbiol Infect Dis.

[B6] Lam SK, Talley NJ (1998). Consensus conference on the management of *Helicobacter pylori *infection. J Gastroenterol Hepatol.

[B7] Laine L, Estrada R, Trujillo M, Knigge K, Fennerty MB (1998). Effect of proton pump inhibitor therapy on diagnostic testing for Helicobacter pylori. Ann Intern Med.

[B8] Dickey W, Kenny BD, McConnell JB (1996). Effect of proton pump inhibitors on the detection of Helicobacter pylori in gastric biopsies. Aliment Pharmacol Ther.

[B9] Marshall BJ (1993). Treatment strategies for *Helicobacter pylori *infection. Gastrenterol Clin North Am.

[B10] Stolte M, Bethke B (1990). Elimination of *Helicobacter pylori *under treatment with omeprazole. Zeitschrift Fur Gastroenterologie.

[B11] Graham DY, Opekun AR, Jogi M, Yamaoka Y, Lu H, Reddy R, El-Zimaity HM (2004). False negative urea breath tests with H2-receptor antagonists: interactions between *Helicobacter pylori *density and pH. Helicobacter.

[B12] Siddiqi S, Hamid S, Rafique G, Chaudhry SA, Ali N, Shahab S, Sauerborn R (2002). Prescription practices of public and private health care providers in Attock District of Pakistan. Int J Health Plann Manage.

[B13] Sturm AW, van der Pol R, Smits AJ, van Hellemondt FM, Mouton SW, Jamil B, Minai AM, Sampers GH (1997). Over-the-counter availability of antimicrobial agents, self-medication and patterns of resistance in Karachi, Pakistan. J Antimicrob Chemother.

[B14] Price AB (1991). The Sydney System: histological division. J Gastroenterol Hepatol.

[B15] Said RM, Cheah PL, Chin SC, Goh KL (2004). Evaluation of a new biopsy urease test: Pronto Dry for the diagnosis of *Helicobacter pylori *infection. Eur J Gastroenterol Hepatol.

[B16] Schnell GA, Schubert TT, Barnes WG, Rupani MK (1998). Comparison of urease, H & E and culture tests for *Helicobacter pylori*. Gastroenterology.

[B17] Logan RP, Walker MM, Misiewicz JJ, Gummett PA, Karim QN, Baron JH (1995). Changes in the intragastric distribution of Helicobacter pylori during treatment with omeprazole. Gut.

[B18] Veldhuyzen van Zanten SJO, Leung V, O'Rourke JL, Lee A (2003). Gastric Transitional Zones, Areas where *Helicobacter *Treatment Fails: Results of a Treatment Trial Using the Sydney Strain Mouse Model. Antimicrob Agents Chemother.

